# Buffalo Academy of Medicine

**Published:** 1909-09

**Authors:** P. W. Vanpeyma


### Buffalo Academy of Medicine.

### Report of the Commission on Schools and School Buildings.

By P. W. VANPEYMA, M. D., Chairman.

AT the time that your committee made its previous report it was decided that its further work lay along the line of our investigation of the efficiency of the ventilating apparatus being installed in the school buildings recently erected and in process of erection. During the early winter accordingly, the Department of Health was requested to order an examination of Schools Nos. 22, 29 and 62. Later the deputy commissioner of buildings also requested a similar examination of the new school buildings, Nos. 41, 44 and 58.

The reports handed in to the department by the city chemist are incomplete and unsatisfactory. In the cases of Nos. 22, 29 and 62, the number of children in the various class rooms is not given. The report says that windows and doors were at times open. The amount of $Co_2$ in the outside air is not given and there are many other important omissions. In the cases of Nos. 41, 44 and 58 the fact that the amount of air was found insufficient it is now claimed was due to the fan not revolving as rapidly as the specifications demand. Another examination is proposed in order that the contractors may obtain their final payment—this being withheld until proof is furnished of satisfactory work.

Of the three blank forms furnished by the department the one devoted to the most important data was not used. The work will have to be done over again next winter with greater care and completeness.

The last few years show increasing attention on the part of the city officials but the whole matter is still in a very unsatisfactory state.

The most important and positively essential requisite is to have an honest and thoroughly competent sanitary engineer employed to plan and supervise the ventilating and heating plants of all public buildings and more especially of the public schools. It is a fact of record on file at the Department of Health that thousands of the pupils of the public schools are daily confined in rooms where the condition, so far as the supply and quality of the air is concerned, is far below that needed to maintain health and vigor.

